# HGSHS-5:G—First results with the short version of the test for the Harvard Group Scale of Hypnotic Susceptibility and a comparison with the full version

**DOI:** 10.3389/fpsyg.2024.1422920

**Published:** 2024-07-31

**Authors:** Nina Zech, Bjoern Riegel, Bjoern Rasch, Burkhard Peter, Ernil Hansen

**Affiliations:** ^1^Department of Anesthesiology, University Hospital Regensburg, Regensburg, Germany; ^2^Private Practitioner, Hohenwestedt, Germany; ^3^Department of Psychology, University of Fribourg, Fribourg, Switzerland; ^4^MEG-Foundation, Wilhelmsthal-Hesselbach, Germany; ^5^Department of Restorative, Preventive and Pediatric Dentistry, School of Dental Medicine, Bern, Switzerland

**Keywords:** hypnotic susceptibility, hypnotic suggestibility, hypnotizability, HGSHS, score distribution, suggestibility groups, normal distribution

## Abstract

**Introduction:**

The HGSHS:A is one of the most commonly used measures of hypnotic suggestibility. However, this test suffers from low feasibility due to a time requirement exceeding 1 h, and from a questionable representation of the normal population. Recently, a short version of HGSHS-5:G was developed and published, and now the first results are available. The scope of this investigation was to verify the assumption of equally positioned and normally distributed scores, resulting in equally sized suggestibility groups in a number of different studies with full or short versions of HGSHS, and to compare the results of the 11-item score with the 5-item score, the latter being calculated from either the full version or the short version test.

**Methods:**

Data from 21 studies with testing for HGSHS were analyzed, 15 using the HGSHS:A full version and six using the HGSHS-5:G short version, for a total of 2,529 data sets. Position and distribution of both the 11-item score and the 5-item score were tested. Linear regression analysis was used to compare the two scores, as well as cross-table and weighted Cohen’s kappa to determine the match of grouping into low and high suggestibility. To evaluate contributing factors to the observed differences in the study results, a multifactorial analysis of variance was performed.

**Results:**

In the different studies, position and distribution of scores, as well as group sizes for low and high suggestibles, varied. All score distributions were found to be non-normal and shifted to the right from the middle score; the shift was more extensive with the 11-item score. The correlation between both scores calculated from full version tests was moderate (*R*^2^ = 0.69), as was the match of suggestibility grouping (κ = 0.58). Studies using the short version involving less student-dominated populations showed sufficient agreement with the full version, but lower scores were caused by an increase in the zero score.

**Conclusion:**

A normal population is not represented in most applications of HGSHS, and grouping into low and high suggestibles varies, mainly due to different positions of score distributions. A direct comparison of full and short versions of HGSHS tested in the same subjects is still missing.

## 1 Introduction

Hypnotizability is the inherent, intra-individual ability of a person to engage in the experience of hypnotic phenomena or to demonstrate them after a hypnosis induction. It also presents as “hypnotic susceptibility” or “hypnotic suggestibility,” and is a widely discussed topic in hypnosis literature (Christensen, 2005; Acunzo and Terhune, 2021; Peter, 2024a,b, this issue). Multiple scales have been designed to gauge levels of hypnotizability for clinical and experimental purposes. They have mainly been developed more than 50 years ago, and it can be discussed, if they suit our current knowledge or if even a next generation of hypnosis scales is needed (Acunzo and Terhune, 2021). Issues of validity and reliability of these hypnotizability scales as measurement instruments and their implementation methods (e.g., group vs. individual, live vs. tape, suggestibility or hypnosis) have been frequently discussed (Woody et al., 2005; Jensen et al., 2017). However, the representativity of these scales has rarely been evaluated. In the beginning, it has been assumed and published that hypnotizability is a trait that is normally distributed in humans: “systematic work has shown that the ability to enter hypnosis is normally distributed in the normal population” (Frankel and Orne, 1976). The bell shape of the normal distribution is indeed found in most normalization samples of the Harvard Group Scale of Hypnotic Susceptibility of Shor and Orne (1962) (e.g., Coe, 1964; Sheehan and McConkey, 1979; Bongartz, 1985; Piesbergen and Peter, 2006). In addition, the impression of a normal distribution results in the assumption of equal-sized groups of low and high suggestibles (LS and HS) on the two sides of the bell curve. In general, those tests are mainly used to group participants into LS and HS for a planned study. The location and the exact distribution of the scores are mostly neglected. However, these characteristics are of utmost relevance for the classification and for the frequent selection of exclusively the highly suggestibles for therapy or research.

Another problem in determining hypnotizability is representativeness. The concept of normal distribution insinuates that the reference population is the “normal population.” Therefore, the selected samples for hypnotizability tests should represent the normal (i.e., the general) population for the results of hypnotizability tests to reference this general population. However, this is often not the case (Peter and Roberts, 2022). Most experimental studies on hypnotic suggestibility have been performed with volunteers. Mainly, they consisted of students, predominantly students of psychology, receiving credits for that. Moreover, in psychology classes today, there is a predominance of female students. With this very restricted test population, a sample-selection bias has to be considered, far from representing the general population (Peter and Roberts, 2022). Nevertheless, a normal distribution of suggestibility scores is commonly observed. But there are exceptions, for instance, data from dentists using hypnosis showed a right-skewed distribution (Wolf et al., 2022).

It has often been noted that the existing hypnotizability tests are not well suited to this task, especially not the Harvard Group Scale of Hypnotic Susceptibility (Shor and Orne, 1962), which is the most common test for hypnotizability (see the recent French standardization study of Brunel et al., 2024). One reason for this article is to revisit these and the above-mentioned problems of the conventional HGSHS and to support this with new facts. The second reason is to present for the first time results obtained with the recently introduced short version of the HGSHS-5:G (Riegel et al., 2021) and to compare them with results from the original long version of the HGSHS:A.

One of the most common tests for hypnotic suggestibility is the Harvard Group Scale of Hypnotic Susceptibility (Shor and Orne, 1962). In its original form, the HGSHS:A consists of a hypnosis induction followed by 12 tasks, namely (1) head falling, (2) eye closure, (3) hand lowering, (4) arm immobility, (5) finger lock, (6) arm rigidity, (7) hands attraction, (8) head shaking inhibition, (9) experience of a fly, (10) eye catalepsy, (11) posthypnotic suggestion, and (12) amnesia. The test takes about 1 h, which is hardly practical for hypnotherapeutic practice or clinical studies, especially for those with patients during a hospital stay. Therefore, recently, a short version has been developed after a thorough analysis of the contribution of the various items (Riegel et al., 2021). Meanwhile, this HGSHS-5:G has been used in a couple of studies in different populations (Zech et al., 2019, 2020, 2022, 2023). Although a direct comparison of the two versions in one and the same test population is still missing, first conclusions can be drawn. For instance, in several studies, no normal distribution of the scores was observed in contrast to the original description.

The article does not undertake to develop, propose, or justify a short version of HGSHS, but presents here the first results of HGSHS-5:G tests available so far and compares them with a separate set of results obtained with the HGSHS full version. The main focus is on location and distribution of the scores. Moreover, we calculated five-point scores from tests with the full version for comparison with scores where only those five items were tested. The aim of this evaluation was to verify the hypothesis that HGSHS testing with either the full or the short version would result in consistently positioned and normally distributed scores as well as equally and consistently sized suggestibility groups. Moreover, our focus was on the reliability of the 5-item score to predict the results of the 11-item score, as well as matching its classification into groups of low and high suggestibility. Observation of any differences in the results of studies or score systems calls for evaluation of contributing factors such as age, gender, and other personal characteristics, as well as test condition parameters. Differences in results and in the impact of various factors might be expected from any shortening of a test, but unexpected changes could also be observed that need consideration when these tests are applied. Nevertheless, this is not a review, and we cannot undertake to assess and discuss in detail all aspects of hypnotizability testing. Instead, the article aims to give additional information on practical aspects of the application of HGSHS in its full or shortened version.

## 2 Materials and methods

### 2.1 Data acquisition and participants

Data from 15 studies using the full version HGSHS:A and six studies using the shortened version HGSHS-5:G were included in the analysis. Study topics and characteristics are shown in Table 1.

**Table 1:** Studies included in the analysis.

Study	HGSHS version	Participants	Study topic	Publication
1	Full	71 students	Repeated exposure to hypnosis on hypnotizability	Rasch, unpublished
2	Full	103 students	Experience and presentation modality and hypnotizability	Rasch and Cordi (2024)
3	Full	148 students	Hypnotic suggestion effects on daytime sleep	Cordi et al. (2014)
4	Full	62 elderly	Hypnotic suggestion effects on sleep in the elderly	Cordi et al. (2015)
5	Full	92 volunteers	Hypnotic suggestion effects on nighttime sleep	Cordi et al. (2020)
6	Full	85 volunteers	Relaxing music and sleep	Cordi et al. (2019)
7	Full	26 students (psychology)	Guided imagery and sleep	Cordi and Rasch (2021)
8	Full	56 volunteers	EEG-derived scores during trance induction	Zech et al. (2023), this issue
9	Full	50 volunteers	Suggestion effects on max. Arm muscle strength	Zech et al. (2019)
10	Full	246 students	Hypnotizability and trance depth	Riegel et al. (2021)
11	Full	100 students	Hypnotherapeutics affect regulation	Riegel et al. (2021)
12	Full	48 students	Hypnotizability and trance depth	Riegel et al. (2021)
13	Full	417 students	Hypnotizability and personality	Riegel et al. (2021)
14	Full	366 volunteers	Hypnotherapy for smoking cessation	Riegel et al. (2021)
15	Full	99 secondary school	Hypnotizability, personality style, and attachment	Peter et al. (2014)
16	Short	45 surgical patients	Suggestion effects on max. Arm muscle strength	Zech et al. (2020)
17	Short	50 volunteers	Suggestion effects on max. Respiratory muscle strength	Zech et al. (2022)
18	Short	57 sport students	Suggestion effects on max. Hand muscle strength	Franzke (2016)
19	Short	276 surgical patients	Effects of suggestions during general anesthesia	Nowak et al. (2020)
20	Short	123 hypnosis meeting	Suggestibility in hypnosis-trained persons	Peter, unpublished
21	Short	15		Rasch, unpublished
	Sum	*N* = 2,529		

HGSHS, Harvard Group Scale of Hypnotic Suggestibility; full version = HGSHS:A; short version = HGSHS-5:G.

### 2.2 Suggestibility tests used in the analyzed studies

The HGSHS:A (Shor and Orne, 1962) is the most used and researched hypnosis scale in the world. It is an adaptation of a group administration with self-report scoring of the original, individually administered, and objectively scored Stanford Hypnotic Susceptibility Scale (SHSS:A) (Weitzenhoffer and Hilgard, 1959). It was used in the German version introduced by Bongartz (1985) with a tape recording of the same author. The 12th item, a highly variable posthypnotic amnesia item, was inconsistently reported in most studies. Therefore, for consistent application, only the results from the first 11 tasks were used in the calculation of scores (Peter et al., 2015).

The HGSHS-5:G is a shortened version of the HGSHS:A, consisting of the motor challenge items 4 (arm immobility), 5 (finger lock), 6 (arm rigidity), 8 (head shaking inhibition), and 10 (eye catalepsy) (Riegel et al., 2021). Available audio tapes were used, one edited from the full version recording of W. Bongartz, and another one recorded by one of the authors (EH).

### 2.3 Suggestibility scores and groups

In the HGSHS full version (HGSHS:A), scores were calculated from performance in 11 tasks (11-item score = 11-IS/HGSHS:A), as well as from the five items used in the short version (5-item score = 5-IS/HGSHS:A). In the HGSHS-5:G short version, scores were calculated from fulfillment of the five included items (5-IS/HGSHS-5:G). The scores in the various studies were tested for normal distribution both analytically (Kolmogorov–Smirnov test) and graphically (histograms). However, the analytical tests are known to be highly dependent on sample size and on the number of possible values (six in case of 5-item score). To consider further influencing factors for grouping and group size of LS and HS, additional measures for characterization of score distributions were introduced. The position of the score distribution was described by the mean score and then by the percentage deviation from the middle, which is 5.5 for the 11-IS, and 2.5 for the 5-IS, respectively. For two-peaked distributions in studies with the shortened version, the portion of subjects with a score result of zero was calculated in addition (% zero score). When using the 11-IS, subjects were assigned to groups of low suggestibility (LS) according to scores 0–3, median suggestibility (MS) for scores 4–7, and high suggestibility (HS) for scores 8–11, respectively. Analogously, using the 5-IS, subjects were assigned to groups LS (scores 0–1), MS (scores 2–3), and HS (scores 4–5), respectively.

### 2.4 Parameters extracted from the studies

Participant-specific parameters in the included studies were recorded and analyzed for their impact on score and group results: age (mean age and age groups), sex (male or female), and occupation (scholar, student of psychology, student of other faculties, employee, pensioner). Because of a reported non-linear relationship with a maximum age effect at 36–55 years (Riegel et al., 2021), three age groups were formed: (“young” = 15–30 y, “middle-aged” = 31–50 y, and “old” = 51–85 y) for evaluation of the impact of age on suggestibility group allocation, and 8 age groups for multifactorial analysis of score position. Study-specific parameters registered were: type of suggestibility test (HGSHS:A, HGSHS-5:G), and study type (hypnosis study, other study, hypnosis training).

### 2.5 Statistical analyses

The presented data were derived from 21 studies with various study designs and purposes (see Sections 2.1 and 3.1) and were combined into a large study population for the first time. For better clarity and visualization of metric data like score or age, histograms were generated and analyzed. In order to describe and compare the position of the score distribution, the mean and the percentage deviation from the theoretical middle of the 11-IS and 5-IS were calculated. Relationships between 11-IS and 5-IS calculated from full version tests, as well as between the therefrom derived suggestibility groups, are presented in cross-tables. Linear regression analysis was performed, and the weighted Cohen’s kappa coefficient was calculated to determine the match of classification into the categories LS and HS. In addition, linear regression analysis was performed to assess the relationship between the full and shortened versions of the HGSHS test.

To evaluate contributing factors for the position of the score distribution (mean score), a multifactorial analysis of variance each for the two scores (11-IS and 5-IS) of the HGSHS full version as well as for the short version as a dependent variable, including sex, age group (in steps by 10 years each), occupation, and study type as independent variables were applied. *Post-hoc* multiple testing for least significant differences (LSD) was used. Thereby, interactions of factors are considered, resulting in adjusted means. Additionally, we tested for multicollinearity, as predictors might correlate. For simplicity and to provide a straightforward interpretation of the effects, no random intercepts for the different studies were considered in our models. The potential impact of contributing factors for the categorical grouping into suggestibility groups, especially the practically relevant proportion of “high suggestibles,” was analyzed by the group sizes (%LS, %HS). A *p* < 0.05 was considered statistically significant for all tests. All analyses were performed with IBM SPSS Statistics, Version 27.

## 3 Results

### 3.1 Differences in biographic data

**Figure 1:**
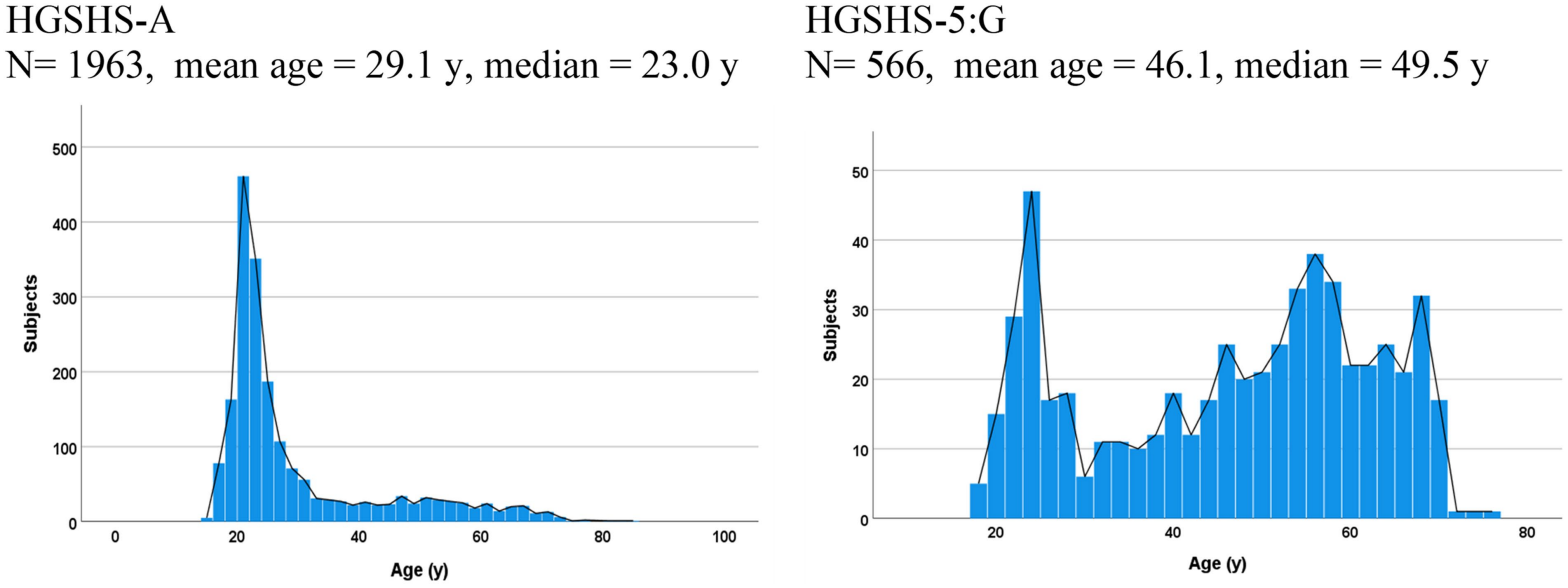
Age distribution in studies with full and shortened versions of HGSHS.

The included studies differed markedly in study objectives and populations (Table 1). Studies #1–15, using the full version of HGSHS:A, were predominantly performed with students in the age distribution shown in Figure 1, whereby 74.0% of participants were of young age (≤30 years). In studies with the shortened version HGSH-5:G, the mean age was higher (Table 2), and the age distribution was bicuspid, with only 24.2% of participants being of young age. In studies #1–15 with HGSHS:A, a higher proportion of women participated (73.1%) than in studies with HGSHS-5:G (54.0%) (Table 2). In latter studies #16–21, participants were volunteers or patients, presenting a mixture of young and elderly persons, students, working people, and retirees. Moreover, studies #16–19 included studies without reference to hypnosis, while study #20 was conducted with participants of a hypnosis meeting and therefore with explicit reference to hypnosis.

**Table 2:** Biographic data and score results of all included studies.

Study #	HGSHS version	*N*	Mean age (years)	Female	11 item score				5-item score				
					Mean	Deviation from middle	LS (%)	HS (%)	Mean	Deviation from middle	Zero score	LS	HS (%)
1	Full	71	21.8	77%	5.70	+4%	15.5	16.9	2.07	−17%	25%	42.3	26.8
2	Full	103	23.3	75%	5.79	+5%	15.5	21.4	2.42	−3%	15%	35.0	29.1
3	Full	146	22.9	100%	7.27	+32%	0.7	47.3	3.22	+28%	15%	15.8	50.0
4	Full	62	67.5	100%	6.42	+17%	12.9	33.9	2.89	+16%	11%	24.2	40.3
5	Full	92	22.8	61%	7.16	+30%	5.4	47.8	2.84	+14%	10%	21.7	37.0
6	Full	85	33.9	64%	6.44	+17%	11.8	36.5	2.82	+13%	14%	25.9	45.9
7	Full	25	22.6	60%	6.44	+17%	8.0	28.0	2.36	−6%	16%	32.0	28.0
8	Full	55	35.1	62%	6.56	+19%	12.7	36.4	2.85	+14%	14%	25.5	45.5
9	Full	48	33.6	55%	6.00	+9%	14.5	29.2	2.56	+2%	12%	27.1	31.3
10	Full	246	24.0	79%	5.93	+8%	13.0	23.2	2.53	+1%	16%	30.1	32.9
11	Full	100	22.9	73%	5.86	+7%	18.0	23.0	2.34	−6%	15%	34.0	28.0
12	Full	48	22.3	73%	6.17	+12%	16.7	31.3	2.58	−3%	12%	25.0	33.3
13	Full	417	22.8	81%	5.85	+6%	13.4	24.0	2.50	±0%	15%	32.6	31.2
14	Full	366	44.2	58%	6.64	+20%	11.7	40.7	2.84	+14%	11%	25.1	40.7
15	Full	99	16.7	57%	5.76	+5%	16.2	24.2	2.72	+9%	5%	14.1	27.3
	All full	1,963	29.1	73.1%	6.24 ± 2.27	+13.4%	12.2 ± 4.6	30.9 ± 9.4	2.66 ± 1.64	+6.4%	13%	27.4 ± 7.3	35.1 ± 7.6
16	Short	45	43.8	56%					2.40	−4%	18%	26.7	22.2
17	Short	50	29.1	60%					1.72	−31%	34%	50.0	14.0
18	Short	57	23.1	63%					1.42	−43%	30%	57.9	7.0
19	Short	276	52.5	49%					1.65	−34%	42%	54.7	21.0
20	Short	123	52.0	n.d.					3.24	+30%	10%	19.5	56.1
21	Short	15	30.8	93%					2.33	−6.8%	20%	40.0	26.7
	All short	566	46.1	54.0%*					2.06 ± 1.80	−18.6%	31%	41.5 ± 15.6	24.5 ± 17.0
	#16–19	428	45.0	54.0%					1.71	−31.6%	37%	47.3 ± 14.1	16.0 ± 7.0

HGSHS, Harvard Group Scale of Hypnotic Susceptibility; full version = HGSHS:A; short version = HGSHS-5:G; * available results.

Theoretical middle of the 11-IS is 5.5, and of the 5-IS is 2.5.

### 3.2 11- and 5-item scores in studies using HGSHS:A

**Figure 2:**
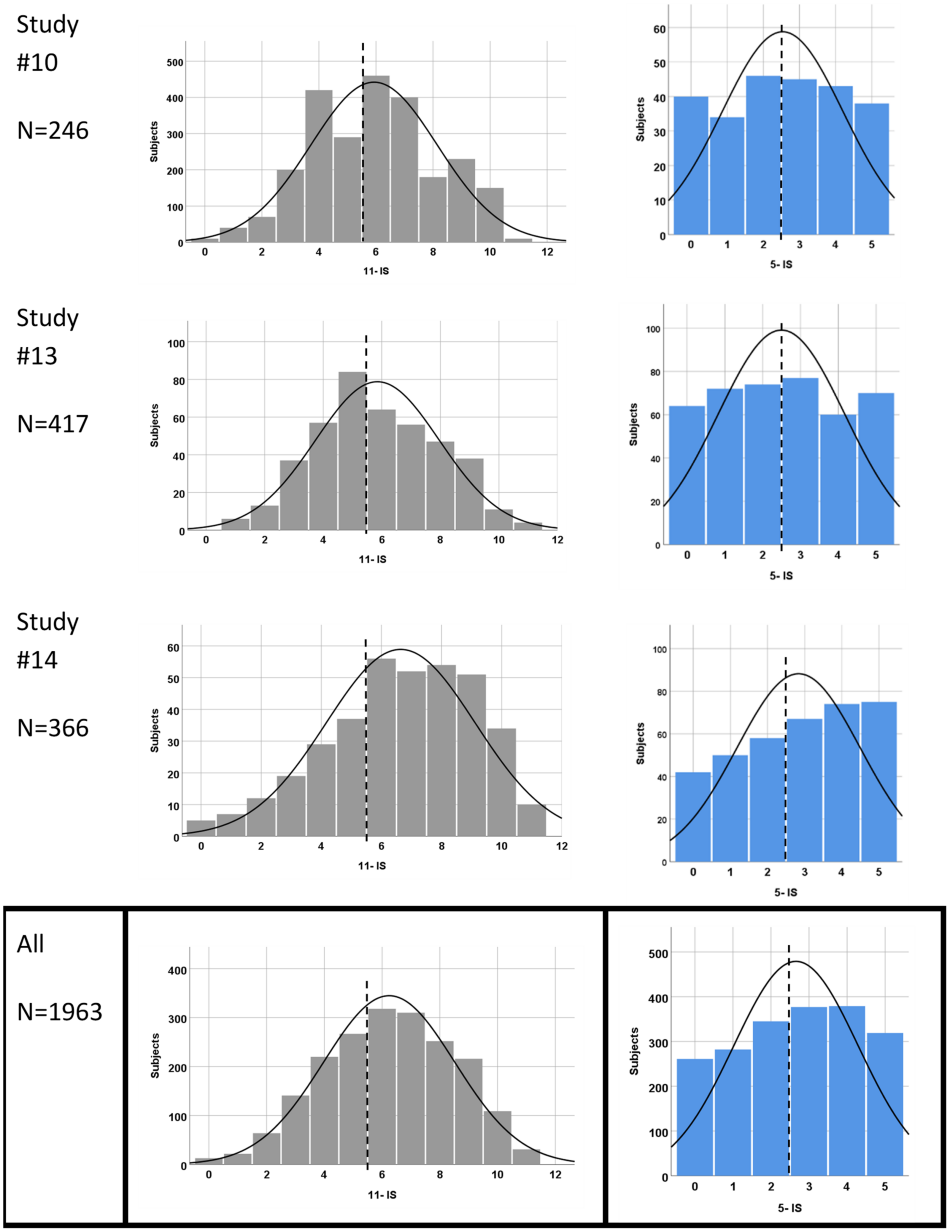
Examples and sum of score distributions from HGSHS: A tests. Studies with *N* > 200 were selected as examples. 11-item scores in gray, and 5-item scores in blue. The dashed line marks the middle of the score system, and the black line represents an assumed normal distribution.

Both scores extracted from HGSHS:A studies were not normally distributed according to the Kolmogorov–Smirnov test, neither in the individual studies nor in their sum. The histograms also showed the deviation from a normal distribution, with the exception of the sum of all 11-IS. Examples are given in Figure 2, where the deviation from the black-lined bell curve is visible. In addition, all score distributions were not centered around the middle (of the score system) but shifted to higher values (Figure 2 and Table 2). Position and distribution of the scales differed between the studies. The deviation of the mean from the middle to higher values ranged from 5 to 32%, averaging 13.4%. Results for the calculated 5-IS/HGSHS:A showed a flatter distribution with a smaller right shift, on average by 6.4% (range: −6 to +28%, see Table 2). A shift toward higher 11-IS values, as in study #3 or #14, is similarly reflected in the 5-IS distribution. The mean score over all studies is 2.66 with a relative standard deviation of 62%, which is higher than 35% in the 11-item score. The average difference in scale distribution between 11-IS and 5-IS was 7 percentage points.

**Figure 3:**
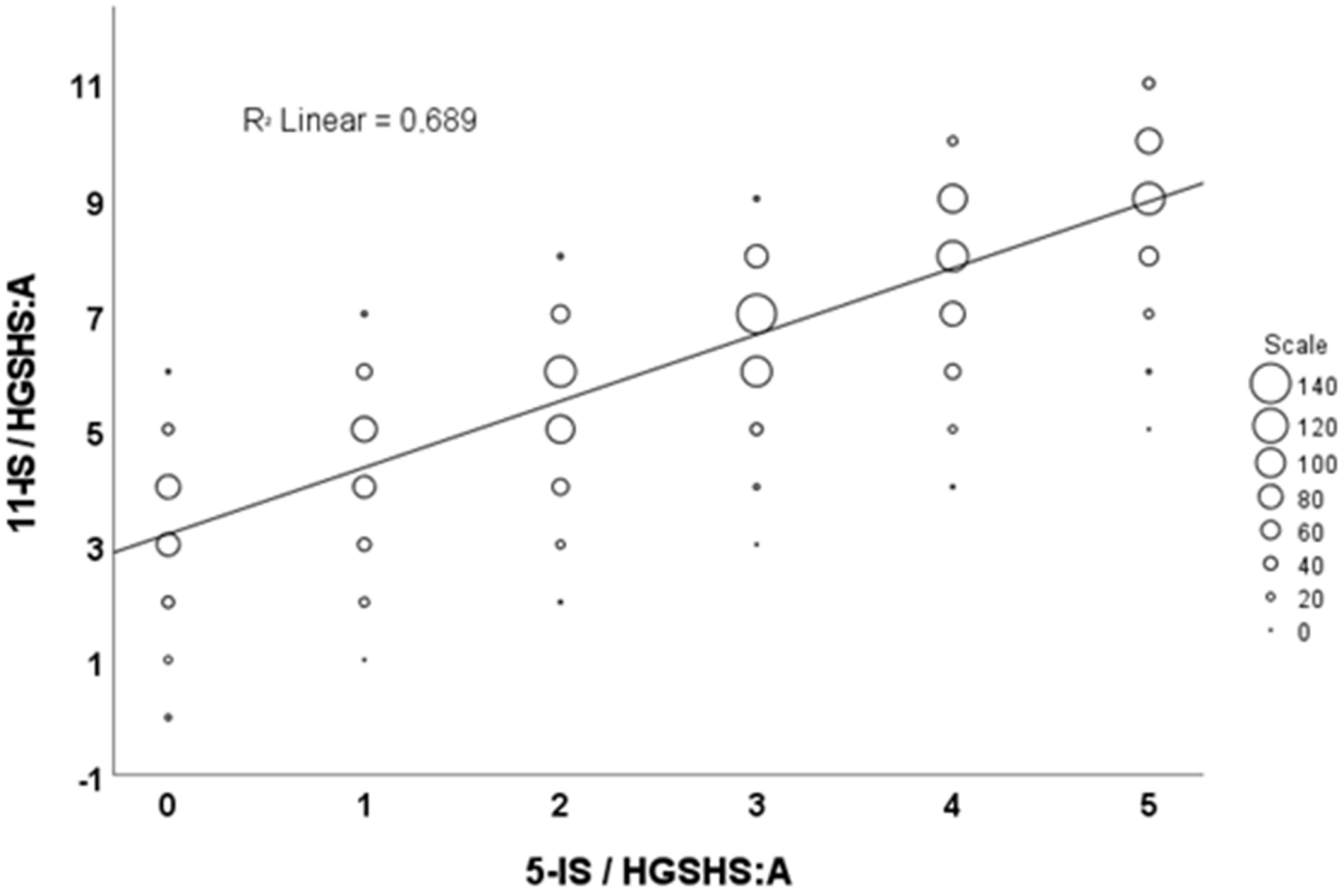
Linear regression analysis of 11-IS and 5-IS from HGSHS:A tests. 11-IS/HGSHS:A = 11-item score derived from HGSHS full test version, 5-IS/HGSHS:A = 5-item score calculated from full version tests.

Evaluation of the relationship between 11- and 5-item scores from HGSHS:A tests revealed that a zero score in 5-IS corresponds to scores of 11-IS in a range of 0–6 with a maximum at 3, and the highest 5-IS score of 5 corresponds to scores in a range of 5–11 with a maximum at 9. The linear regression analysis is presented in Figure 3 and resulted in a coefficient of determination *R*^2^ of 0.689 for the prediction of 11-ISs from 5-IS values.

### 3.3 Match of classification into low and high suggestibles

In the studies using the HGSHS:A grouping according to the 11-IS is asymmetrical, with 12.2% LS and 31.0% in HS (Table 3). Moreover, classification into the groups of low (LS) and high (HS) suggestibility varied between 0.7 and 18.0%, or 16.9 and 47.8%, respectively, in these studies (Table 2). In addition, grouping according to the calculated 5-IS is not symmetrical, with 27.7% LS and 35.6% HS in a range from 14.1 to 42.3%, or 26.8 to 50.0%, respectively. From the cross-table of suggestibility grouping, a weighted Cohen’s kappa of 0.578 is derived for the match of the two scoring systems. The table shows that 84% of test subjects rated HS according to the 11-IS are also high suggestibles in 5-IS, and 73% of those high in 5-IS are also highs according to 11-IS. Of the participants rated LS in 11-IS, 88% fell into the same suggestibility group in the 5-IS analysis, but only 39% of the LS in the 5-IS group were rated LS by the 11-IS analysis.

**Table 3:** Cross-table of suggestibility groups according to 11 or 5 items.

Weighted kappa = 0.578		5-IS/HGSHS:A			Total
		Suggestibility group			
		Low	Medium	High	
11-IS/HGSHS:A	Low	211	29	0	240
					12.2%
Suggestibility group	Medium	332	597	186	1,115
					56.8%
	High	0	96	512	608
					31.0%
Total		543	722	698	1,963
		27.7%	36.8%	35.5%	100%

11-IS, 11-item score; 5-IS, 5-item score; HGSHS:A, full version of Harvard Group Scale of Hypnotic Suggestibility.

### 3.4 Scores and suggestibility groups in studies using HGSHS-5:G

**Figure 4:**
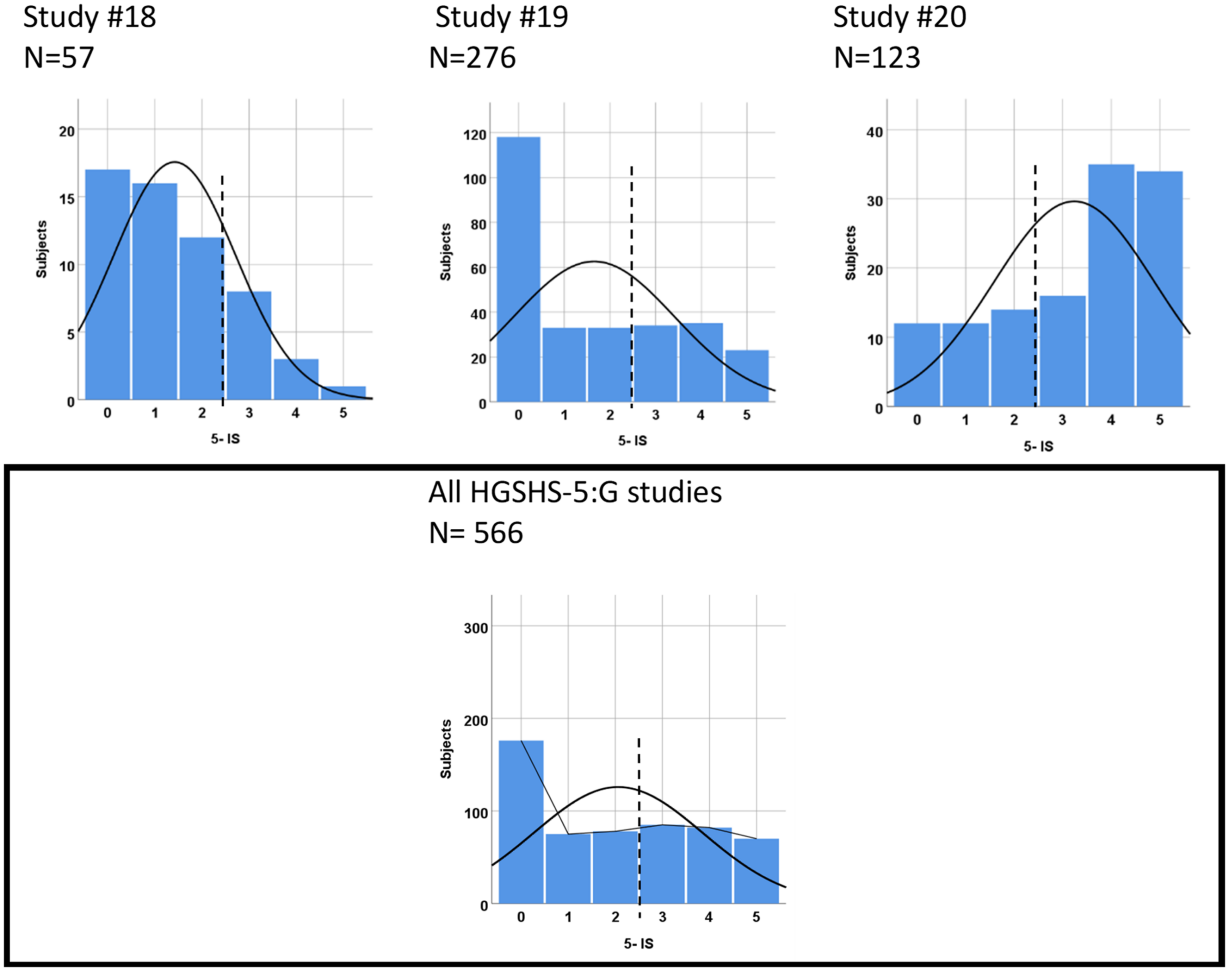
Score distributions from HGSHS-5:G tests: examples and sum of all six studies.

The 5-ISs in studies #16–21 with the HGSHS-5:G were positioned considerably further to the left, i.e., shifted to lower suggestibility scores (Table 2 and Figure 4), by −18.6% from the middle (2.5). While the only study of participants from a hypnosis meeting (study #20) showed a marked right shift (a mean of 30% from the middle), the four studies of volunteers or patients (studies #16–19), laypersons regarding hypnosis, revealed a strong left shift (a mean of −31.6% from the middle). Of the latter, three presented two-peaked distributions as well as the sum of the HGSH-5:G studies. The portion of participants with a score of zero adds up to more than 30%, in strong contrast to 5-ISs calculated from HGSH:A studies that contained only 13% with a zero score. Accordingly, a lower proportion of study participants were rated HS, on average 24.5%, and the sizes of the suggestibility groups were disproportionate, with 41.5% LS (Table 2).

### 3.5 Influencing factors for mean score and suggestibility classification

Personal and study characteristics documented in the included studies and therefore available for an evaluation of their potential influencing factors were age, gender, occupation, and study type (e.g., “hypnosis study,” HGSHS:A or HGSHS-5:G, relation of participants to hypnosis). We tested these factors for multicollinearity and found variance inflation factors (VIF) between 1.03 and 1.92 for the predictors (age group, gender, study type, and occupation). Therefore, every factor was included in further analyses.

For the analysis of the key target variable “mean score,” we used a multifactorial analysis of variance. In addition, for the practically relevant target “suggestibility group,” other considerations are necessary.

Age: Statistical significance of age effects was found in multifactorial analyses only for the 11-IS (*p* = 0.02), not for the HGSHS-5:G (*p* = 0.09) or the 5-IS/HGSHS:A (*p* = 0.78), respectively. For adjusted means and *p* according to *post-hoc* analyses, see Table 4. For the 11-IS, significant differences were found, especially for young and especially old participants; however, the latter group only had an *n* of 3. The sizes of suggestibility groups differed between the three age groups, both according to 11-IS or 5-IS (Table 5). The unequal size of LS and HS was more pronounced with the 11-IS.

**Table 4:** Multifactorial analysis of influencing factors on score position with *post-hoc* multiple LDS testing; the brackets describe significant differences (according to *post-hoc* analyses for contributing factors).

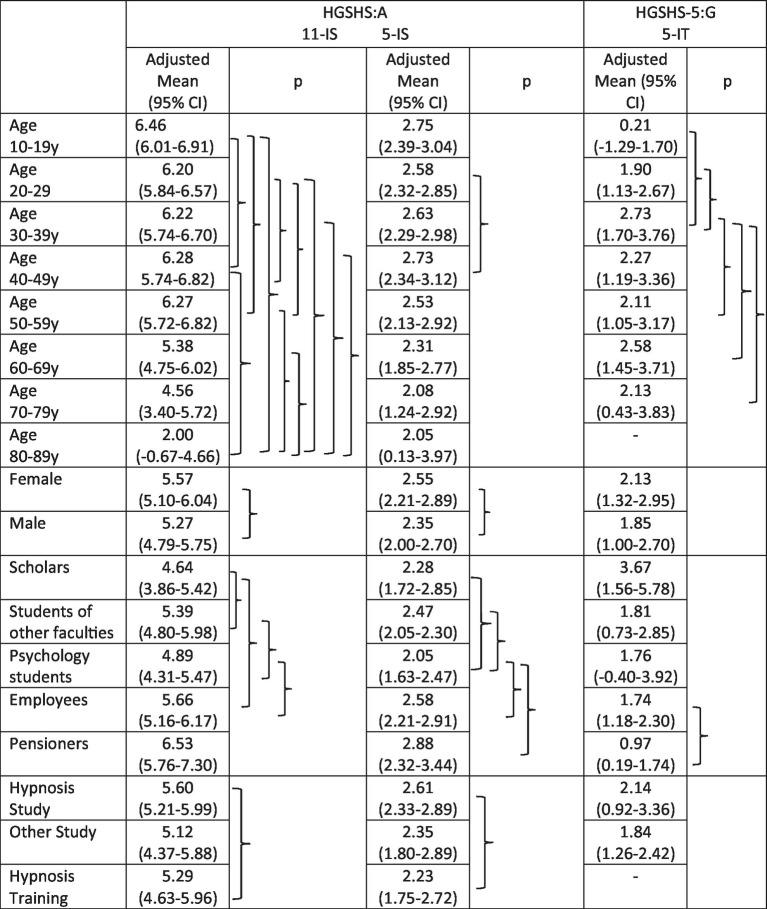

HGSHS:A, full version of Harvard Group Scale of Hypnotic Suggestibility; 11-IS, 11-item score; 5-IS, 5-item score.

**Table 5:** Variables of suggestibility group allocation.

	11-IS/HGSHS:A		5-IS/HGSHS:A	
	LS (%)	HS (%)	LS (%)	HS (%)
All	12.2	31.0	27.7	35.6
Young (15–30)	12.5	28.3	29.2	33.3
Middle-aged (31–50)	10.4	40.1	22.6	45.5
Old (51–85)	12.9	36.5	24.5	37.8
Female	11.1	31.1	26.6	36.4
Male	15.2	30.7	30.6	33.4
Students from psychology	13.4	25.3	33.1	29.6
Students from other faculties	10.7	32.4	30.0	40.6
Employees	11.5	39.2	22.5	40.4

LS, low suggestibility; HS, high suggestibility; 11-IS/HGSHS:A, 11-item score derived from HGSHS full test version; 5-IS/HGSHS:A, 5-item score calculated from full version tests.

Gender: Females and males differed significantly in position and distribution of both 11-IS and 5-IS from full version tests in the multifactorial analysis of variance (for adjusted means and *p*, see Table 4). The influence of gender was not significant using the short version, even if the difference seemed more pronounced. While HS group size differed only slightly between females and males, the ratio of HS to LS was higher in females than in males (Table 5). This imbalance was more pronounced with the 11-IS than with the 5-IS.

Occupation: In the multivariate analysis of variance correcting for effects of the other factors, statistically significant differences were observed between scholars, students, employees, and pensioners, both with 11Is and 5-IS of full version tests (Table 4), not for the short version. Differences of highest significance were found between students of psychology and other faculty members, as well as employees. With the short version HGSHS-5:G, scores were found to be statistically different between pensioners and employees. While the portion of test subjects categorized as HS was highest in employees according to 11-IS or 5-IS, respectively, for LS it was highest in students of psychology (Table 5). Highest weighted kappa representing conformity of 11-IS and 5-IS for suggestibility group classification results were identified for employees (κ = 0.62).

Study type: For different references of participants to hypnosis, given by declaration as “study of hypnosis” or not, or history of training in hypnosis, the multifactorial analysis showed no effect on achieved scores (Table 4) or suggestibility grouping. Merely, the subgroup of participants with a kind of training in hypnosis showed significant effects for the 11-IS and 5-IS in the *post-hoc* analyses, respectively (Table 4).

## 4 Discussion

### 4.1 Characteristics of the HGSHS:A

Most published results of hypnotic suggestibility or hypnotizability testing with HGSHS:A confirm a normal distribution. Based on this normal distribution, the subjects are usually categorized into three groups of low, medium, and high suggestibles (LS, MS, and HS), and it is assumed that the LS and HS groups are distributed symmetrically on both sides of the distribution curve by about 10–25%. In contrast, in our evaluation, no normal distribution of scores was found in any of the studies in either scoring system. Moreover, the present evaluation reveals a marked variation in position and form of the 11-item scale distribution when analyzing the included 15 studies in detail (see Figure 2 and Table 2). For representation of this shift in the position distinct from any “skewness,” the “deviation from the middle” was introduced deliberately for comparison to the 5-item score discussed later and might be useful for comparison to other hypnotic suggestibility scales as well.

Doubts about HGSHS results being representative of the normal population have been raised before, especially since predominantly psychology students were tested in hypnosis research (Peter and Roberts, 2022). The difference in score results that we have observed between psychology students and students of other faculties raises interesting questions regarding whether the bias only relates to age. Moreover, our evaluation of studies that include participants with educational backgrounds in addition to significant age differences underscores the potential for variation that comes with it.

A practical disadvantage of HGSHS:A is the time requirement of more than 1 h that has led to the request for a shortening (Terhune and Cardena, 2016) and resulted in the development of a short version, the HGSHS-5:G, with a test time of only 25 min (Riegel et al., 2021).

### 4.2 Characteristics of the HGSHS-5:G

The present study represents the first evaluation of available data from that short version HGSHS-5:G. However, before we discuss the results of tests performed with this short version, we look at the 5-item scores that have been extracted from the full 11-score version, the HGSHS:A. The calculation of 5-IS from the evaluated studies with the full version HGSHS:A shows a flatter score distribution, as is to be expected when reducing the number of factors included in the analysis (Figure 2). Deviation from a “normal distribution” is more frequent, and the positions of the score distribution are shifted less from the middle to the right, i.e., toward higher scores. With a linear regression coefficient of *R*^2^ = 0.69 (Figure 3), the relationship between 11-IS and 5-IS calculated from the same HGSHS:A tests is only moderate.

Unexpectedly, results from tests with the short version HGSHS-5:G, as far as yet available, show quite a distinct picture. The mean score in the studies is found to be markedly shifted to lower hypnotic suggestibility (except in study #20 on participants in a hypnosis meeting). However, most of the score histograms are two-peaked rather than normally distributed, and the left shift can be explained by a disproportionate increase in test participants who scored zero points. Only the study with strongly hypnosis-interested people showed a clear right shift, and the one with sports students showed a clear left shift. This high proportion of zero-point results indicates a marked difference between 5-IS derived from full and short versions of HGSHS tests.

### 4.3 Hypnotizability and the HGSHS

The notion of the “normal distribution of hypnotizability” is widespread and persists even in recent publications: “Furthermore, multiple studies have shown a generally normal distribution of hypnotizability scores with most individuals scoring in a moderate range (Coe, 1964; Bongartz, 1985; Piesbergen and Peter, 2006), and a small proportion scoring in the low or very high range” (Elkins, 2024, p. 1), even if this author admits immediately afterwards: “Several past studies have suggested that hypnotizability may be a multifactorial construct. However, it is unknown as to whether hypnotizability is best accounted for as being multifactorial or as a general factor with subcomponents.” Hilgard et al. (1961) was already concerned with this topic when describing the standardization attempts of the Stanford Hypnosis Susceptibility Scale (SHSS) (Weitzenhoffer and Hilgard, 1959), where originally (Hilgard et al., 1961) a bimodal distribution had been found. The issue of bimodality eventually concerned other researchers, such as Balthazard and Woody (1989). Based on a factor analysis, Woody et al. (2005) determined four distinguishable subscales as the “building blocks of hypnotic response,” and, finally, Sadler and Woody (2021) provided a general historical overview and prospect of multicomponent theories of hypnotizability. The question of whether hypnotizability has taxonomic or dimensional properties (Balthazard and Woody, 1989; Oakman and Woody, 1996; Reshetnikov and Terhune, 2022), whether latent patterns could be found specifically in highly hypnotizable individuals (Kihlstrom, 2015; Terhune, 2015), and whether a general “G-factor” correlated with minor co-factors underlies hypnotizability (Zahedi and Sommer, 2022; Brunel et al., 2024; Zimmerman et al., 2024) have been studied. These recent results are appealing because they support the basic idea of hypnotizability as a fundamental, albeit variable, human “trait,” which has been assumed for almost 250 years (Peter, 2024a,b, this issue). However, it has been evaluated since around the middle of the last century, that large parts of the variance, are explained by other mediating and moderating co-factors, i.e., well-studied social-psychological, socio-cognitive, and contextual variables which we refer to as “state” variables. This should not be confused with the term “altered state of consciousness,” which was much discussed in the older hypnosis literature. Instead, we would describe hypnotic trance as a transient state dependent on socio-cognitive determinants. In addition to hypnotizability, the factor of suggestibility must always be considered, which also plays an important role in human communication and interaction outside of hypnosis (e.g., Braffman and Kirsch, 1999; Zahedi and Sommer, 2022). So, it is safe to say that our conventional scales are far from measuring just hypnotizability or hypnotic suggestibility as a global and uniform human trait which is normally distributed—even if this is repeatedly claimed. The widely used measures have different properties that result in the loss of valuable information, including binary scoring and single-trial sampling, and hinder their utility, such as the inclusion of suboptimal suggestion content (Acunzo and Terhune, 2021).

The present evaluation cannot dissolve the ongoing discussion on hypnotizability and its testing but can contribute some new aspects. The results question the normal distribution of HGSHS, both in its full or shortened version of testing, as well as the equal size of the derived suggestibility groups.

### 4.4 Influencing variables

With the high variation in score position and distribution observed, a question arises regarding the reasons for such large differences between studies, score systems, and test systems. Of course, the wide range of target groups in the included studies contributes to the diversity of results while bringing hypnotic suggestibility testing much closer to a “normal population” than experimental conditions that involve predominantly psychology students. By analyzing the different characteristics of the study populations, comprising age, gender, occupation, and attitude toward hypnosis, we were able to test for their effects on study results. Additionally, the difference in scores and test system can have an impact too.

A dependency of hypnotic suggestibility on age is well known (Page and Green, 2007), although this correlation is expectedly not linear (Riegel et al., 2021). High hypnotic suggestibility in children is followed by lower scores in young adults. After a maximum of around the age of 45, suggestibility declines again (Morgan and Hilgard, 1973). With the differentiation of eight age groups, several results of the present evaluation indicate an age effect, especially for the 11-IS. However, in the multifactorial analysis, the statistical significance is lost for the five-item versions. A reason might be the confounding simultaneous influence of multiple factors with overlap, e.g., the variable age with features like occupation distinguishing between scholars, students, employees, and pensioners. The effect of gender on the results of tests for HGSHS (Költö et al., 2014) is confirmed in this study, except for the short version HGSHS-5:G. Obviously, the variance of test results can be explained only to a limited extent by commonly monitored biographic features like age and gender, and even with additional variables like occupation (important to represent a normal population) and attitude toward hypnosis (Green and Lynn, 2011).

With regard to the latter, interest in, or knowledge of hypnosis has to be considered (Capafons et al., 2008). Hypnotherapists describe a personality profile that differs significantly from that of people who are not interested in hypnosis and reveal a characteristic trait. Hypnosis practitioners had high scores in the personality style intuitive/schizotypal, which led to the term “homo hypnoticus” (Peter and Böbel, 2020). These individuals, as well as patients successfully treated with hypnotherapy, are convinced and expect themselves to respond to hypnotic suggestions and consequently reach higher scores. Students of psychology who depend on credits from study participation are also ready and willing to perform properly and fulfill the tasks. In addition, the response of test subjects may vary depending on whether they are participating in a “hypnosis study.” Interestingly, the present evaluation shows higher scores for students of other subjects than for students of psychology when all 11 items are considered instead of only five items. Moreover, the highest scores were observed for pensioners (in 11-IS/HGSHS:A and 5-IS/HGSHS:A) or scholars (in 5-IS/HGSHS-5:G) (see Table 4). This is again in contrast to a representation of the normal population and the common tests of students. Neither the expected familiarity of psychology students with hypnosis, nor an association of the HGSHS test with a “study about hypnosis,” nor a prior experience with hypnosis turned out to be a significant determinant for higher suggestibility scores. An exception was observed in a study of participants in a hypnosis meeting (study #20), which has been repeated in the meantime, and the results are anticipated to be available soon.

Differences in the test system have to be considered as well. Often, the HGSHS test is described as 12 tasks set after a hypnosis induction. Actually, however, the first two items, namely head falling and eye closure, are initiated during the hypnosis induction and should therefore be considered to be part of it. Moreover, the execution of the following tasks may also contribute to depth of hypnosis by repeating words like “as you relax more and more.” Therefore, any shortening of the HGSHS:A by reducing the tasks may have an impact on the depth of trance. It should be noted that even the short version HGSHS-5:G was delivered in two versions: one including item #1 (head falling), although it was excluded from scoring. Especially the rise in score zero in some of the HGSHS-5:G applications could be due to a lower depth of hypnosis. Moreover, the change of the 11-item scoring to the 5-item scoring involves selection of, and limitation to the five motoric challenge items of HGSHS:A. The exclusion of the perceptual and cognitive items results in different people responding differently to the full and short versions of hypnotic suggestibility testing, thereby fulfilling the requested tasks to a different extent. So, the most significant difference between the original HGSHS:A and the shortened HGSHS-5:G version is that the original HGSHS:A still contains all four different types of items that Woody et al. (2005) extracted by factor analysis (direct motor, motor challenge, perceptual-cognitive, and posthypnotic amnesia), while the HGSHS-5:G consists only of motor challenge items. According to Zahedi and Sommer (2022), the outcome of these challenge suggestions can significantly predict the outcomes of both the direct-ideomotor and cognitive-perceptual suggestions but not vice versa, which means that this group of challenge items is of particular importance. They refer to the criterion of involuntariness, an important characteristic of hypnotic experience (alongside evidence, i.e., when the hypnotic experience is felt as real) (Peter, 2015, 2024a,b, this issue). In conclusion, our evaluation shows an unexpected high variation in position and distribution of suggestibility scores in different studies using different scoring systems. Primarily, hypnotizability has been seen as a trait compatible with a normal distribution. Our observations of a wide variation in position and distribution of suggestibility scores and the failure to explain these differences with trait factors like age, gender, or occupation that contribute to the variability draw attention to hypnotic suggestibility as a non-trait but “state” condition, in the sense that social-psychological and socio-cognitive theorists understand it. For instance, the different results of studies #12 and #13 (with a mean of 6.17 vs. 5.85 and an HS group size of 31% vs. 24%), both performed on students and with similar scope, cannot be explained by age or gender effects alone. In older studies, the trait characteristic of hypnotizability was often emphasized, for example, via its heritability (Morgan, 1973) or its stability over a period of 25 years (Piccione et al., 1989). This view was called into question, and alternatively, it was claimed and proven that state differences such as motivation, personal relationships, expectations, or demand characteristics amply researched by social-psychological and socio-cognitive hypnosis researchers (e.g., Spanos, 1991; Spanos and Coe, 1992; Kirsch, 2001; Lynn et al., 2019) play a much more important role, quite apart from the fact that situational and contextual conditions may be significant. Although some authors have shown that such factors are not very important for high suggestibles (Perry and Sheehan, 1978), we suspect that, at least for the medium suggestible ones, there are major differences between testing them in a university classroom or in a clinic before an operation, as was the case with many of our participants that have been tested under such different conditions. Doubts about the dominant trait nature of hypnotic suggestibility, i.e., susceptibility to suggestions, also stem from the low impact of susceptibility scoring on the efficacy of hypnotherapeutic interventions in psychotherapy (Green and Lynn, 2000) or medicine (Montgomery et al., 2011). This is in line with the mindset and viewpoint of Milton Erickson that hypnosis is mainly a matter of interpersonal relationship (Erickson, 1952; Erickson et al., 1976), which is also in line with initial findings from biochemical hypnosis research (e.g., Varga, 2021). Finally, with regard to our results, one must ask: Does hypnotizability as a trait in the form of an intra-individual variable exist at all and is it different from the inter-individual variable of suggestibility, as Peter (2024a,b) claims, or does hypnotizability in its essence actually consist only of social-psychological and socio-cognitive variables, as Lynn et al. (2019) reaffirmed? Would Bernheim have been right when he said: “*Il n’y a pas d’hypnotisme, il n’y a que de la suggestibilité*” (“There is no hypnotism, there is only suggestion”) (Bernheim, 1917, p. 47). With regard to our results, however, the very pragmatic question is relevant: Are the known scales, especially the HGSHS in long or short form, even capable of measuring these two variables, hypnotizability and suggestibility, in a reasonably differentiated way? Despite the promising results of Zahedi and Sommer (2022) and Brunel et al. (2024), we still do not see a clear answer to this question.

### 4.5 Suggestibility groups

The major practical purpose of tests for hypnotic suggestibility is classification into suggestibility groups, especially to select high suggestible persons (HS) for therapy or a study. The present re-evaluation of 15 studies reveals marked inequality in LS and HS group size, and high variation in the suggestibility group sizes for different studies (Table 2). Therefore, the assumed symmetry of HGSHS score distribution and the reliability of suggestibility grouping are to be questioned. The inequality of group sizes of LS and HS is not only caused by deviation from normal distribution and skewness. We recognized the position of the score distribution as a major determinant. For representation of a shift in the position, the “deviation from the middle” was introduced deliberately. Even a normal distribution of scores can lead to unequal groups of LS and HS in case of a shift. This phenomenon originates from the group definition (0–3 for LS and 8–11 for HS) that is symmetrical to the score (11) but not to the position of the score distribution. The mean deviates from the middle of the score (see Table 2). The consideration of this aspect turned out to be especially valuable for comparison with the 5-item score and might be useful for comparison to other hypnotic suggestibility scales as well.

Grouping according to 5-IS in the same studies using the HGSHS full version was also asymmetrical, however, with a more similar group size of LS and HS. The relationship between suggestibility grouping according to 11-IS or 5-IS and the predictability of one from the other is moderate (weighted kappa = 0.58). Finally, in studies using the short version HGSHS-5:G, the ratio of group size of HS and LS is turned around to a dominance of low suggestible subjects. This corresponds to the left shift of the score distribution and the increased number of test subjects with a score of zero. With the exception of the study on hypnosis meeting participants (#20), this indicates that application of the HGSHS-5:G might result in selection of a markedly reduced group of high suggestibles appropriate, for instance, to be included in a hypnosis study. It turns out that the highly variable selection into suggestibility groups is affected by factors such as age, gender, and occupation (see Table 5). However, the most prominent is the dependency on the position of the suggestibility scale. This parameter, which has been rarely analyzed or considered to be dominant, increases the number of HS with a right shift of the curve, or otherwise diminishes it, with considerable practical significance for therapy or research.

### 4.6 Study limitations

This represents a retrospective analysis of available data from studies using HGSHS:A or HGSHS-5:G. Of course, the appropriate study design for a comparison of the full version to the short version would include both tests to be applied to the same subjects. Furthermore, no detailed investigation of contributing variables was possible because of the limited factors reported in the included studies. Even potentially important factors like attitude toward and experience with hypnosis were not clearly defined and monitored for a reliable analysis. We did not use a random intercept for different studies because such a procedure might have compromised our statement: After all, we basically wanted to show that applying one and the same test can lead to different results under different conditions. And this is precisely what should be taken into account whenever this test is used. Nevertheless, this study comprises one of the largest samples of HGSHS:A results and represents the so far only detailed report of available HGSHS-5:G results. In spite of several limitations, initial conclusions can be drawn that may guide further application of both tests and a general discussion of the evaluation of hypnotizability.

## Data availability statement

The original contributions presented in the study are included in the article/supplementary material, further inquiries can be directed to the corresponding author.

## Ethics statement

The studies involving humans were approved by the local ethics committee of the University Hospital Regensburg. The studies were conducted in accordance with the local legislation and institutional requirements. Written informed consent for participation was not required from the participants or the participants’ legal guardians/next of kin in accordance with the national legislation and institutional requirements.

## Author contributions

NZ: Data curation, Formal analysis, Writing – original draft, Writing – review & editing. BRi: Data curation, Project administration, Supervision, Writing – review & editing. BRa: Data curation, Methodology, Supervision, Writing – review & editing. BP: Conceptualization, Data curation, Formal analysis, Methodology, Project administration, Writing – original draft, Writing – review & editing. EH: Conceptualization, Data curation, Formal analysis, Methodology, Writing – original draft, Writing – review & editing.
